# Heart Failure is an Independent Risk Factor for Incident Hip, Proximal Humerus, and Wrist Fractures in a High-Risk Older Population

**DOI:** 10.1007/s11606-025-10092-w

**Published:** 2026-01-26

**Authors:** Amanda J. Chang, Alan S. Go, Malini Chandra, Laura D. Carbone, Howard A. Fink, Susan M. Ott, Joan C. Lo

**Affiliations:** 1 Department of Adult and Family Medicine, Kaiser Permanente Oakland, Oakland, CA USA; 2 Division of Research, Kaiser Permanente Northern California, Pleasanton, CA USA; 3 Department of Health Systems Science, Kaiser Permanente Bernard J. Tyson School of Medicine, Pasadena, CA USA; 4 Division of Rheumatology, Department of Medicine, J. Harold Harrison, MD. Distinguished University Chair in Rheumatology, Medical College of Georgia at Augusta University, Augusta, GA USA; 5 Edward Hines, Jr. Veterans Administration Hospital, Hines, IL USA; 6 Department of Medicine, University of Minnesota, Minneapolis, MN USA; 7 Geriatric Research Education and Clinical Center, VA Healthcare System, Minneapolis, MN USA; 8 Department of Medicine, University of Washington School of Medicine, Seattle, WA USA

**Keywords:** bisphosphonate, heart failure, osteoporosis, treatment

## INTRODUCTION

Heart failure (HF) is a growing public health problem among older adults. Beyond cardiovascular, mortality, and quality of life consequences, studies suggest that HF increases osteoporosis independent of shared risk factors such as older age, menopause, smoking, and diabetes.^1–3^ Adults with HF may have excess risk of hip and humerus fractures,^2–4^ with higher associated mortality.^5^ While adults with recent-onset HF may have lower bone mineral density than those without HF, bone density differences, osteoporosis risk factors, and some comorbidities do not fully explain the increased risk for osteoporotic fractures, which may be multifactorial.^1^

In this study, we examined the association of HF and incident hip, proximal humerus, and wrist fractures in a high-risk population of adults who initiated osteoporosis therapy with bisphosphonate drugs.

## METHODS

This retrospective study included Kaiser Permanente Northern California members aged 65–84 years who initiated oral bisphosphonate therapy in 2010–2019 (index date).^6^ Those treated with other osteoporosis drugs and those with multiple myeloma, secondary metastatic cancer, selected bone disorders, or receipt of kidney dialysis/transplant were excluded. Prevalent HF was ascertained using ≥ 1 hospital diagnosis or ≥ 3 outpatient diagnoses within five years before index. Incident hip fracture (hospital diagnosis) and proximal humerus and distal radius/ulna (wrist) fractures (hospital/outpatient diagnosis) were ascertained during three years of follow-up using *International Classification of Diseases (ICD-9/10-CM)* diagnosis codes (excluding fractures in the first year if a same-site fracture diagnosis occurred before index and within one year of the incident fracture diagnosis). Baseline covariates included age, self-reported race, ethnicity, smoking, diabetes, rheumatoid arthritis, prior fracture, and body mass index (Table 1). Continued bisphosphonate therapy was based on ≥ 80% adherence (≥ 50–90% in sensitivity analysis) for each 3-month interval of follow-up.

**Table 1:** Baseline Characteristics Among Men and Women Who Initiated Osteoporosis Therapy

	OVERALL N = 60,892	WOMEN N = 50,244	MEN N = 10,648
Age, mean ± SD (years)	73.4 ± 6.0	72.7 ± 6.0	76.6 ± 5.0^a^
Age group (years)			^a^
65–74	34,098 (56.0%)	30,864 (61.4%)	3234 (30.4%)
75–84	26,794 (44.0%)	19,380 (38.6%)	7414 (69.6%)
Race and ethnicity			^a^
Non-Hispanic White	39,984 (65.7%)	32,630 (64.9%)	7354 (69.1%)
Black	1844 (3.0%)	1570 (3.1%)	274 (2.6%)
Hispanic	7189 (11.8%)	6080 (12.1%)	1109 (10.4%)
Asian or Pacific Islander	10,988 (18.1%)	9191 (18.3%)	1797 (16.9%)
Other or unknown	887 (1.5%)	773 (1.5%)	114 (1.1%)
Body mass index category^b^			^a^
< 20.0 kg/m^2^	4969 (8.3%)	4366 (8.8%)	603 (5.7%)
20.0 to < 25.0	22,651 (37.7%)	18,680 (37.7%)	3971 (37.6%)
25.0 to < 30.0 (overweight)	20,185 (33.6%)	16,007 (32.3%)	4178 (39.5%)
≥ 30.0 (obesity)	12,261 (20.4%)	10,437 (21.1%)	1824 (17.3%)^a^
Current smoking^c^	4859 (8.0%)	4041 (8.0%)	818 (7.7%)
Prior fracture^d^	21,263 (34.9%)	17,549 (34.9%)	3714 (34.9%)
Diabetes mellitus^e^	13,027 (21.4%)	10,226 (20.4%)	2801 (26.3%)^a^
Rheumatoid arthritis^e^	1232 (2.0%)	1026 (2.0%)	206 (1.9%)
Heart Failure^f^	2948 (4.8%)	2032 (4.0%)	916 (8.6%)^a^

^a^ p < 0.05 (all p < 0.001) comparing men vs women. ^b^ BMI based on the closest measurement within the two years prior to or on index (1.4% missing). ^c^ Current smoking was ascertained based on information within the five years prior to or on index. ^d ^Prior fracture was based on qualifying diagnosis codes within the five years prior to or on index. ^e ^Diabetes mellitus and rheumatoid arthritis were based on two qualifying diagnoses within the five years prior to or on index. ^f ^Heart failure was based on ≥ 1 hospital diagnosis or ≥ 3 outpatient diagnoses within the five years prior to or on index

Fracture incidence was compared by HF status using the log-rank test. The association of HF and incident fracture was examined using Cox proportional hazards regression, adjusting for baseline covariates, continued osteoporosis therapy, and an interaction term for baseline HF status and sex. These analyses were repeated with Fine-Gray regression models, accounting for death as a competing risk. Adjusted hazard ratios (aHR) are reported with 95% confidence intervals (CI).

## RESULTS

Among 60,892 adults (82.5% women, 65.7% non-Hispanic White, 18.1% Asian/Pacific Islander, 11.8% Hispanic, and 3.0% Black) who initiated bisphosphonate therapy, one-third had prior fracture (34.9%). Men were more likely to be age ≥ 75 years (69.6% versus 38.6%) and have diabetes (26.3% vs 20.4%), and less likely to have obesity (17.3% versus 21.1%) than women (Table 1), but current smoking prevalence was similar (7.7% vs 8.0%).

During up to three years follow-up, the unadjusted incidence (per 1000 person-years, 95% CI) of hip, proximal humerus, and wrist fractures was 6.0 (5.6–6.4), 4.4 (4.1–4.8), and 7.1 (6.6–7.5) for women and 8.4 (7.4–9.5), 2.7 (2.2–3.4), and 3.0 (2.4–3.7) for men, respectively. For each skeletal site, cumulative fracture incidence was higher among those with versus without HF (p < 0.001).

**Figure 1:**
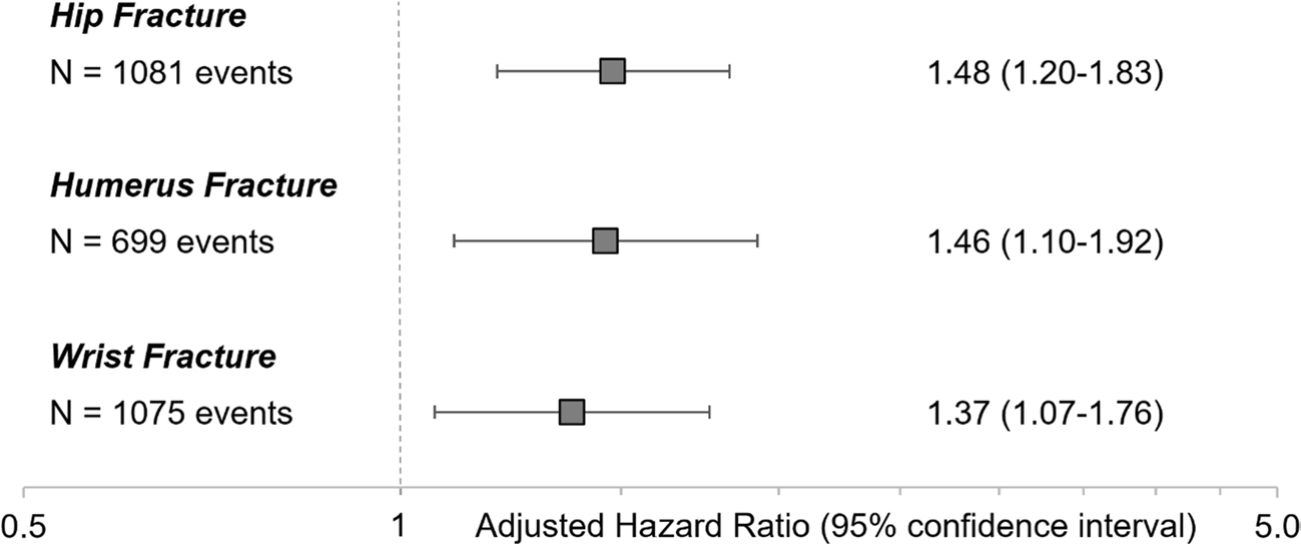
Multivariable association of heart failure and incident major osteoporotic fracture among adults who initiated osteoporosis therapy. Separate multivariable Cox proportional hazard models were conducted for each fracture outcome, adjusting for age, sex, self-reported race and ethnicity, prior fracture, current smoking status, diabetes, rheumatoid arthritis, body mass index (category), and continued osteoporosis therapy during follow-up. There was no significant interaction between heart failure and sex. Adjusted hazard ratios were unchanged when examined using Fine Gray regression models with death as a competing risk. Results were similar/unchanged when using lower (≥ 50%, ≥ 70%) or higher (≥ 90%) adherence to define treatment continuation.

In multivariable analyses adjusting for potential confounders, HF was independently associated with increased risk of hip (aHR 1.48 [1.20–1.83]), proximal humerus (aHR 1.46 [1.10–1.92]), and wrist fracture (aHR 1.37 [1.07–1.76]), with no significant interaction by sex (Fig. 1). Results were unchanged in sensitivity analyses, using ≥ 50% adherence criteria for bisphosphonate continuation.

## DISCUSSION

In a diverse, contemporary population of older adults who initiated osteoporosis therapy, adults with HF had 40–50% higher risk of several types of non-vertebral osteoporotic fracture than those without HF. Building on earlier studies of European and North American populations unselected for osteoporosis,^1–5^ our findings demonstrate that HF remains an independent risk factor for fracture even among higher risk populations who initiate osteoporosis therapy. Our cohort had large representation of US Asian and Hispanic adults, populations that were previously understudied.

Key limitations include lack of data on HF subtype, severity, and pharmacotherapy, frailty/functional status, kidney function, other osteoporosis risk factors, and bone density. Our analyses may have underestimated total osteoporotic burden. Nonetheless, our findings draw attention to HF as an important risk factor for fracture among older adults initiating osteoporosis therapy. In addition to pharmacologic treatment, falls prevention, nutrition, smoking cessation and lifestyle interventions,^7^ identifying modifiable, HF-specific mechanisms contributing to excess fracture risk may support more optimal management of adults with HF. This may be especially important as the US population ages and there is greater intersection of skeletal and cardiovascular health.

## Data Availability

The data that support the findings of this study are available from the corresponding author upon reasonable request. Due to privacy and ethical considerations, access to the data may be restricted and subject to approval by the appropriate institutional review boards.
